# Addressing Lithium Deficiency in Anode‐Free Lithium Metal Batteries: Design Principles and Supplementation Strategies

**DOI:** 10.1002/smsc.202500254

**Published:** 2025-07-16

**Authors:** Maolin Sun, Kai Tang, Zhichuan J. Xu

**Affiliations:** ^1^ School of Materials Science and Engineering Nanyang Technological University 639798 Singapore

**Keywords:** anode‐free lithium metal batteries, cathode additives, electrolyte additives, lithium supplementation, overlithiation

## Abstract

Anode‐free lithium metal batteries (AFLMBs) are now considered as a promising next‐generation energy storage system due to their exceptional energy density and compatibility with existing lithium‐ion battery manufacturing infrastructure. However, their practical deployment is hindered by severe capacity degradation, primarily caused by the irreversible consumption of lithium. This perspective explores how lithium supplementation and recovery strategies can address these challenges by shifting focus from conventional structural engineering to chemical compensation mechanisms. Recent advances are systematically categorized into three main approaches: cathode overlithiation, cathode additives, and electrolyte‐based supplementation. For each strategy, the underlying mechanisms, representative materials, and electrochemical performance are critically evaluated. Particular attention is given to lithium storage capacity, decomposition potential, electrochemical compatibility, and byproduct management. The interdependence between lithium compensation methods and electrode/electrolyte design is also examined to clarify their cooperative or competing roles within full‐cell configurations. In addition, strategies for recovering inactive lithium, including dead lithium reactivation and solid electrolyte interphase reconstruction, are discussed as complementary pathways. By comparing the advantages and limitations of these approaches, this perspective highlights key material design principles and provides practical insights for advancing AFLMB systems with high‐energy density and extended cycling stability.

## Graphical abstract

The lithium supplementation strategy is employed to address the capacity loss in battery systems. This perspective focuses on various lithium compensation strategies in anode‐free lithium metal batteries (AFLMBs), emphasizing their mechanisms, compatibility, and lithium storage efficiency, and further provides insights for advancing the AFLMBs with enhanced energy density and extended cycling stability.

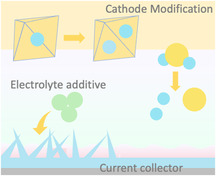

## 1 Introduction

The escalating demand for sustainable energy infrastructure has intensified research into efficient energy conversion and storage technologies. Among them, electrochemical energy storage systems have garnered significant attention, enabling applications from grid‐level stabilization to portable electronics and electric vehicles.^[^ ^1^, ^2^, ^3^ ^]^ Lithium‐ion batteries (LIBs) have long dominated this field due to their high operating voltage, long cycle life, and negligible memory effect.^[^ ^4^, ^5^, ^6^, ^7^, ^8^, ^9^ ^]^ However, despite continued optimization efforts, their practical energy density remains limited to ≈250 ${\text{Wh kg}}^{-1}$, approaching the theoretical ceiling and restricting further development in high‐energy applications.^[^ ^10^, ^11^, ^12^, ^13^ ^]^

**Figure 1:**
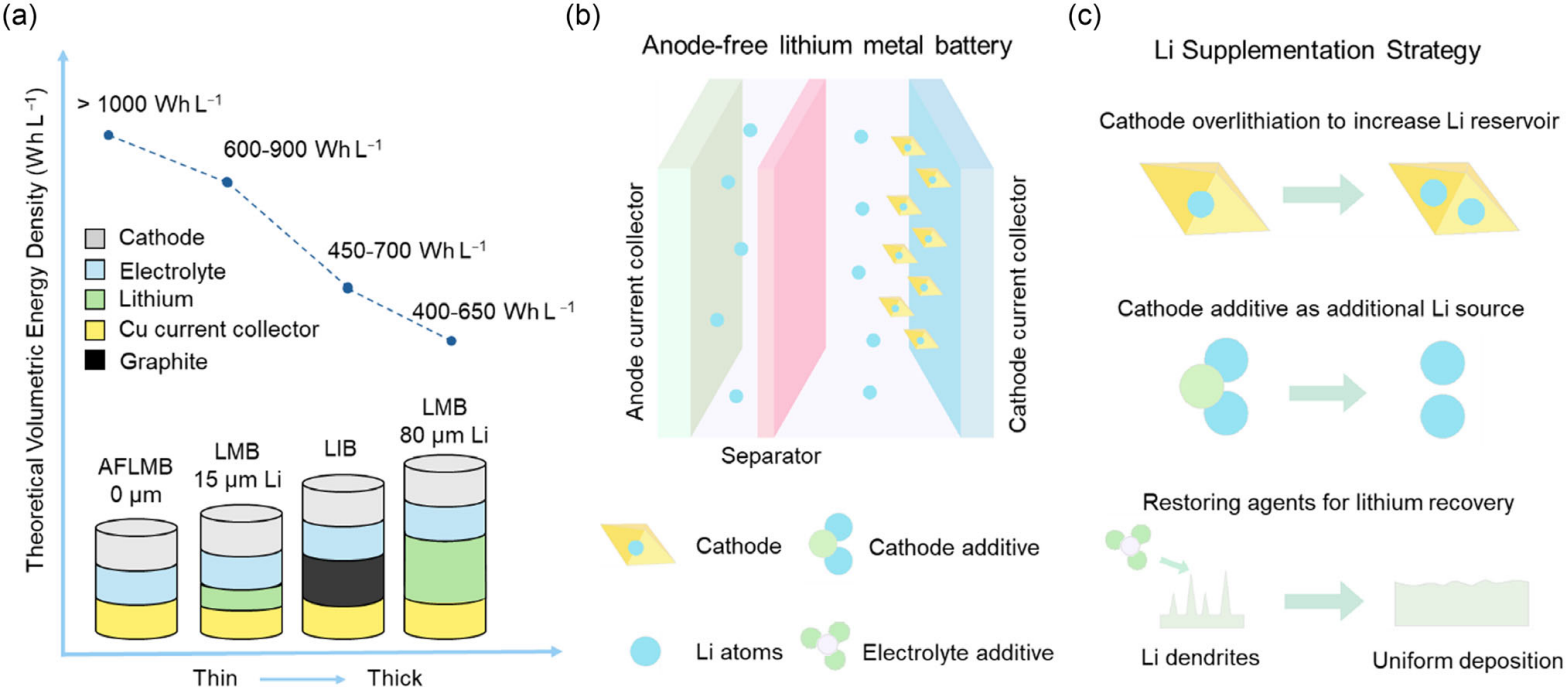
a) Energy density advantage of AFLMBs compared to conventional LIBs. b) Schematic illustration of an anode‐free lithium metal battery. c) Potential strategies for lithium storage and restoration.

To overcome this constraint, anode‐free lithium metal batteries (AFLMBs) have been proposed as a promising alternative.^[^ ^14^, ^15^ ^]^ By utilizing a fully lithiated cathode paired with a bare copper current collector as the anode, AFLMBs eliminate the need for excess lithium metal (**Figure** **1**a,b).^[^ ^16^, ^17^ ^]^ During the initial charging cycle, lithium ions are extracted from the cathode and deposited onto the surface of the current collector. This configuration offers several advantages: 1) The absence of excess lithium or graphite reduces both the volume and weight of the cell, enabling higher energy density.^[^ ^18^ ^]^ 2) All electrode components remain in a fully discharged and less reactive state during assembly, which improves safety. 3) AFLMBs are structurally similar to LIBs and are thus compatible with current manufacturing infrastructure, facilitating their potential scale‐up and commercialization.^[^ ^19^, ^20^ ^]^

Despite these benefits, AFLMBs face critical cycling stability challenges. The high reactivity and considerable volume change of lithium during cycling promote the cracking of the solid electrolyte interphase (SEI). Once disrupted, the exposed anode surface undergoes continuous parasitic reactions with the electrolyte, leading to repeated SEI reconstruction and irreversible consumption of active lithium. Additionally, the anode current collector, which often lacks sufficient lithiophilicity, promotes nonuniform lithium deposition. This results in lithium dendrite formation and the accumulation of electrically isolated dead lithium, further exacerbating irreversible lithium loss.^[^ ^15^, ^21^, ^22^, ^23^, ^24^, ^25^ ^]^ Given that AFLMBs operate without excess lithium, these losses have a more detrimental impact than in conventional batteries, typically resulting in poor cycling stability and short lifespan.^[^ ^26^, ^27^, ^28^, ^29^ ^]^

In response to these challenges, research efforts have been dedicated to modifying AFLMBs through anode current collector engineering and electrolyte design. Both approaches primarily improve Coulombic efficiency (CE) by directly regulating anode deposition behavior or suppressing parasitic reactions. However, their limited effectiveness has not led to substantial improvements in cycle life. Consequently, lithium supplementation technologies targeting irreversible capacity loss have been developed for AFLMBs. The lithium supplementation technologies involve directly introducing supplemental lithium sources into the battery before or during the early cycles, helping to preserve capacity and extend cycle life.^[^ ^30^, ^31^, ^32^, ^33^ ^]^ Due to the common issue of lithium deficiency shared by LIBs and AFLMBs, some supplementation techniques originally developed for LIBs can also be adapted for AFLMB systems. In addition to lithium supplementation, recent studies have highlighted the importance of restoring inactive lithium, including electrically isolated or passivated lithium that forms during cycling. These recovery strategies aim to convert “dead lithium” back into active material, further supporting long‐term stability.

In this perspective, we present a summary of recent advances in lithium supplementation and restoration strategies tailored for AFLMBs. Three principal strategies are identified to mitigate irreversible lithium loss: cathode overlithiation, cathode additives, and electrolyte additives (Figure 1c). For each method, we delve into the operational principles, assess the role of material selection, and evaluate the effectiveness of these strategies in improving the cycle life and efficiency of AFLMBs. Key performance metrics such as lithium supply capacity, decomposition potential, and structural compatibility are considered to offer a comprehensive comparison. Collectively, this perspective provides insights into material design principles and strategic directions that may facilitate the development of high‐energy density AFLMB systems with extended cycling stability.

## 2 Strategies for Lithium Supplementation and Recovery

### 2.1 Overlithiation of Pristine Cathode Material

**Figure 2:**
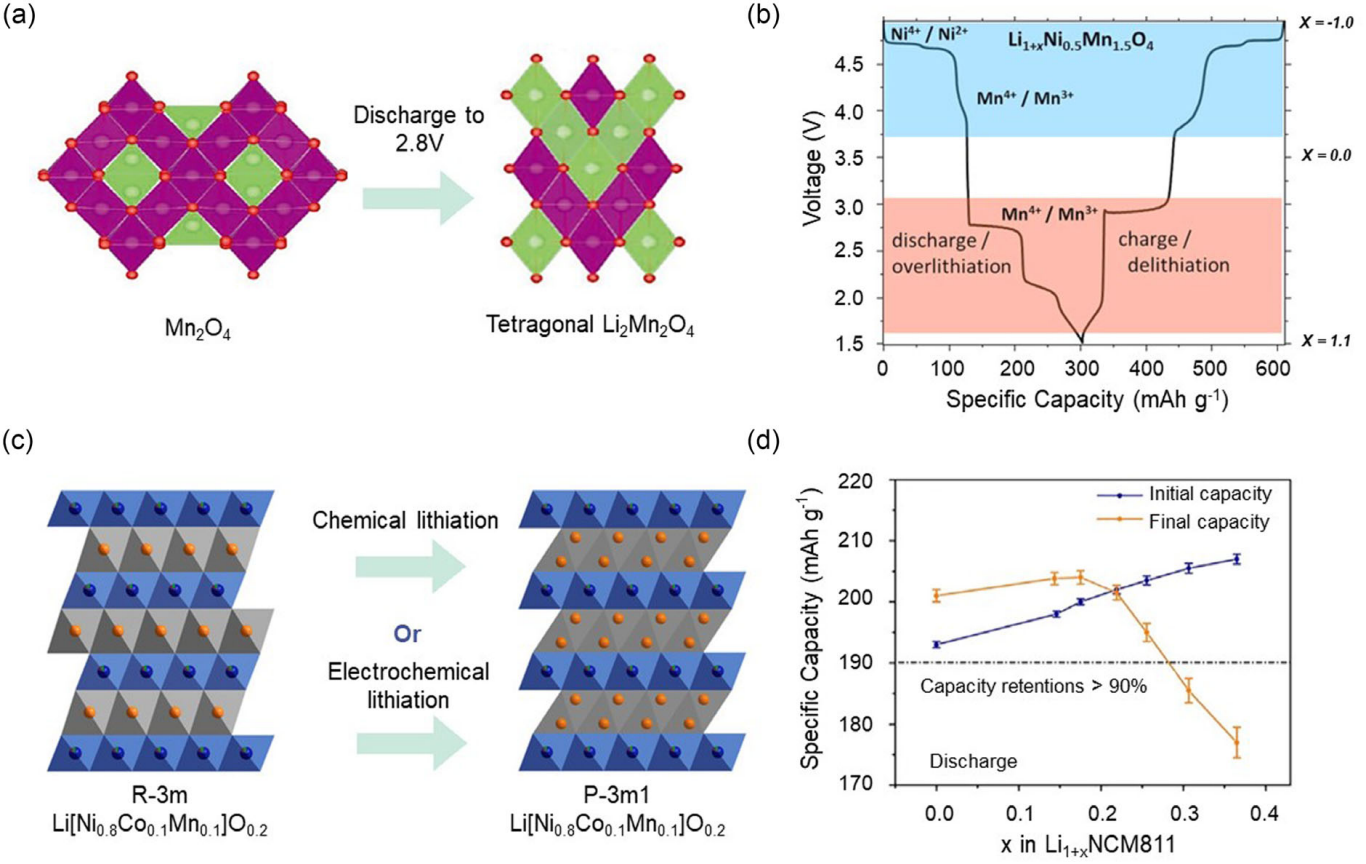
a) Schematic illustration of structural transformations in spinel‐structured electrode materials during overlithiation. Reproduced with permission.^[^ ^36^ ^]^ Copyright 2020, Advanced Energy Materials. b) Voltage profile of an LiNMO half‐cell, which is charge/discharge between 4.95 and 1.5 V. Reproduced with permission.^[^ ^38^ ^]^ Copyright 2019, Electrochemical Society, Inc. c) Schematic illustration of structural transformations in NCM811 electrode materials during overlithiation. d) Voltage profile of NCM811 half‐cell and Li_1.2_NCM811 half‐cell, which charge/discharge between 4.3 and 2.6 V. Reproduced with permission.^[^ ^43^ ^]^ Copyright 2021, Wiley‐VCH.

Overlithiation of cathode materials involves the incorporation of excess lithium, which can be released during the initial charging process to mitigate irreversible lithium capacity losses. The primary advantage of overlithiation is that it does not generate any inactive substances within the cathode. After the initial charging process, the material that releases excess lithium can still function as cathode. Tarascon et al. first proposed the method of utilizing chemical overlithiation of LiMn_2_O_4_ to mitigate the irreversible capacity loss in their study.^[^ ^34^ ^]^ Research indicates that the spinel structure of Mn_2_O_4_ is capable of intercalating one ${\text{Li}}^{+}$at 4.1 V to form LiMn_2_O_4_, and can further accommodate a second ${\text{Li}}^{+}$ at 2.8 V, resulting in the formation of Li_2_Mn_2_O_4_. Aravindan et al. further explained its mechanism.^[^ ^35^ ^]^ They indicated that, within the spinel phase of Mn_2_O_4_, the first lithium preferentially intercalates into the tetrahedral sites, leading to the formation of the cubic phase of LiMn_2_O_4_. Subsequently, a second lithium intercalates into the octahedral sites, and with the cubic phase undergoes a transformation into a tetragonal phase, resulting in a volume change of ≈3% (**Figure** **2**a).^[^ ^36^ ^]^ Consequently, while Li_2_Mn_2_O_4_ is not suitable for direct application as a cathode material, it can effectively release lithium during initial charging to mitigate irreversible capacity loss. During actual electrochemical overlithiation processes, the maximum value of x in Li_1+x_Mn_2_O_4_ reaches 0.75, which corresponds to an approximate lithium supply of 100 mAh g^−1^. The transient volume change in the first charging has an ignorable impact on the stability of the electrode.

To avoid continuous formation of Li_2_Mn_2_O_4_ in subsequent cycles, the cycling potential was elevated to 3.5–4.5 V, which may influence the complete release of lithium in LiMn_2_O_4_ at tetrahedral sites. To address this problem, the typical high‐voltage cathode material Ni was doped into the spinel phase of Mn_2_O_4_ to form Li_1+x_Ni_0.5_Mn_1.5_O_4_ (Li_1+x_NMO), thereby enhancing the deintercalation potential of lithium at these sites. The voltage profile of Li_1+x_NMO is shown in Figure 2b. Following Ni doping, the deintercalation potential of lithium at tetrahedral sites reached 4.7 V. Research indicates that for Li_1+x_NMO, the maximum value of *x* is 0.93, corresponding to an approximate lithium supply of 135 mAh g^−1^.^[^ ^37^, ^38^, ^39^ ^]^ Lin et al. utilized the Li_2_NMO as a cathode material in AFLMBs.^[^ ^40^ ^]^ The assembled Li_2_NMO|Cu pouch cell achieved an energy density of 367 Wh kg^−1^ and maintained a capacity retention rate of 88% over 50 cycles. Although significant advancements have been made in overlithiation methods for spinel‐structured cathode materials, the tendency of Mn^3+^ to undergo disproportionation and dissolution leads to a relatively low cycle life for these materials, thereby limiting their applications.

The overlithiation of Li[Ni_x_Co_y_Mn_1−x−y_]O_2_ (NCM) has also received extensive attention to address its issue of low cycle life.^[^ ^41^, ^42^ ^]^ Lin et al. investigated the overlithiation process of Li[Ni_0.8_Co_0.1_Mn_0.1_]O_2_ (NCM811) cathode and successfully synthesized a core‐shell structured Li_1+x_[Ni_0.8_Co_0.1_Mn_0.1_]O_2_ (Li_1+x_NCM811) using both chemical and electrochemical techniques.^[^ ^43^ ^]^ At 1.8 V, a doubling of the lithium content was introduced into NCM811, resulting in a structural transformation. Lithium migrated from octahedral voids to tetrahedral voids, which diminished the diffusion capability of lithium within Li_2_NCM811 and induced a volume change of 6.8% during the lithium insertion process (Figure 2c). Therefore, Li_2_NCM811 can serve as a lithium supply during the initial charging process instead of directly being applied as a cathode material. Although the theoretical maximum value of x in Li_1+x_NCM811 is 1, substantial volume changes occur when x exceeds 0.4, significantly compromising cathode stability (Figure 2d). A pouch AFLMB utilizing Li_1.37_NCM811 as its cathode achieved an energy density of 447 ${\text{Wh kg}}^{-1}$ and demonstrated a capacity retention rate of 84% after 100 cycles.

### 2.2 Cathode Additives for Lithium Supplementation

In contrast to overlithiation, cathode additives impose less stringent compatibility on the pristine cathode composition. A single additive can often be applied across multiple cathode materials, highlighting a key advantage of this strategy. However, the performance of different types of cathode additives strongly depends on their decomposition potentials and the surrounding electrolyte environment, which may hinder their universal applicability across diverse cathode systems.

Among various cathode additives, Li_2_O exhibits exceptional overall performance and has been the subject of extensive research.^[^ ^44^, ^45^ ^]^ Due to the large potential hysteresis, its release of lithium could be predominantly irreversible. However, during the decomposition process, there exists a relatively high‐energy barrier, which results in elevated actual decomposition potential. Sun et al. investigated the use of transition metal/Li_2_O nanocomposites as additives for lithium supplementation in cathodes.^[^ ^46^ ^]^ The nanocomposites were synthesized by mixing molten Li_2_O with transition metal oxides under an argon atmosphere, where a conversion reaction (1) took place.

(1) $$M_{x}O_{y}+2\text{yLi}\to xM+{\text{yLi}}_{2}O$$

(2) $$M+2{\text{Li}}_{2}O\to {\text{MO}}_{2}+4{\text{Li}}^{+}+4e^{-}$$

**Figure 3:**
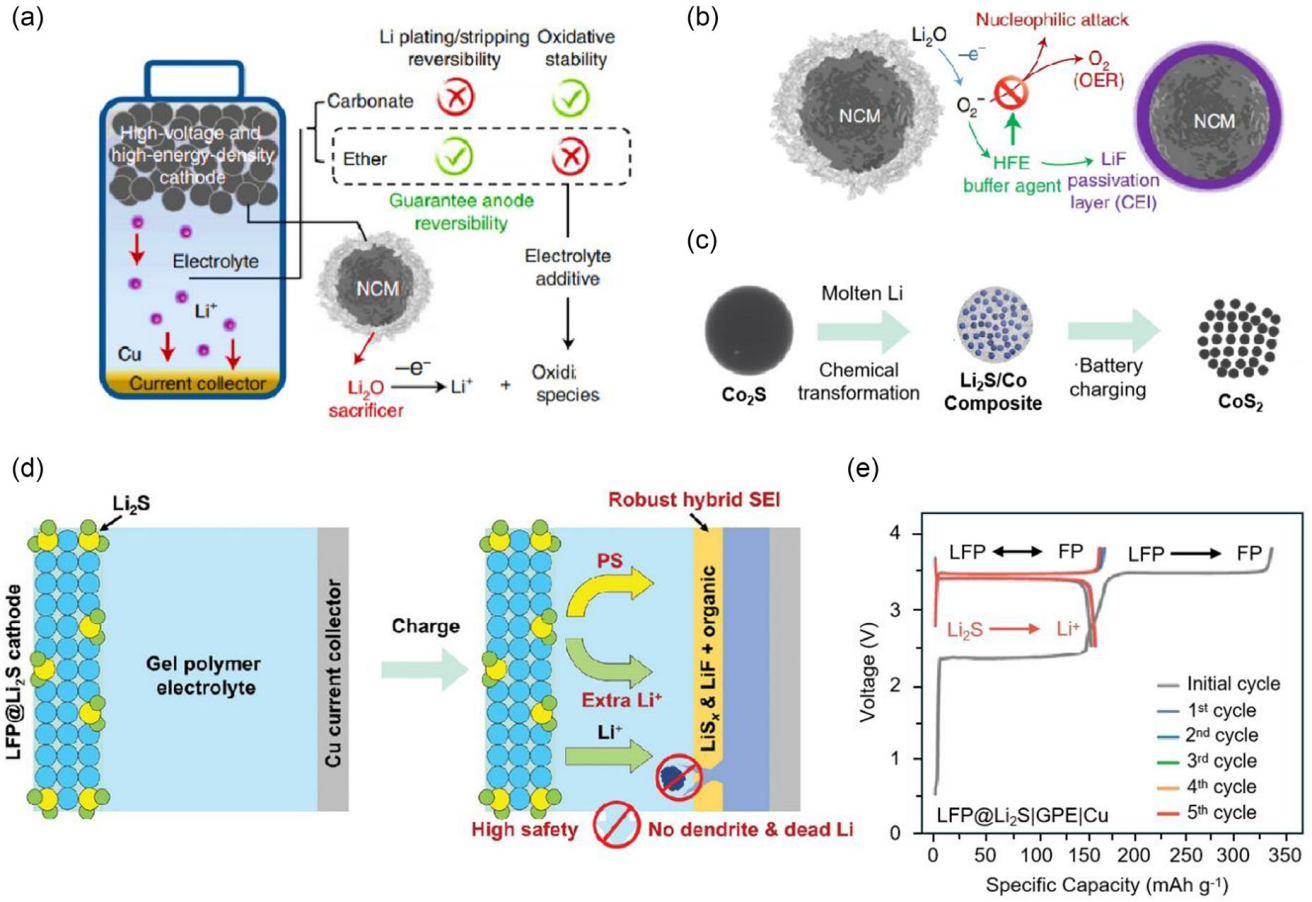
a) Schematic illustration of the operating principle of Li_2_O cathode additive in a modified ether‐based electrolyte system. b) Schematic illustration of the formation mechanism of the LiF protective layer on the surface of the cathode material. Reproduced with permission.^[^ ^47^ ^]^ Copyright 2021, Springer Nature. c) Schematic of the chemical synthesis of Li_2_S/metal composite and the electrochemical extraction of lithium during the battery charge process. Reproduced with permission.^[^ ^51^ ^]^ Copyright 2016, Wiley‐VCH. d) Voltage profile of AFLMB assembled with HFE added electrolyte and NCM811@Li_2_O cathode, which charge/discharge between 4.4 and 2.8 V. e) Voltage profile of AFLMB assembled with GPE and LFP@Li_2_S cathode, which charge/discharge between 3.8 and 2.5 V. Reproduced with permission.^[^ ^52^ ^]^ Copyright 2023, Wiley‐VCH.

As shown in the reaction (2), the incorporation of transition metals altered the lithium release mechanism from Li_2_O, effectively mitigating the release of O_2_. Although the catalysis of transition metals effectively mitigates the release of O_2_, the unavoidable increase in the ineffective mass of the cathode consequently results in a reduction in energy density. Therefore, a more optimal strategy is to diminish the adverse effects of O_2_ on the battery system while ensuring that the additive undergoes complete decomposition. Abouimrane et al. combined Li_2_O with a layered Li_2_MnO_3_‐LiMO_2_ composite cathode material.^[^ ^44^ ^]^ This incorporation significantly enhanced its electrochemical performance. Due to electrolyte decomposition or redox shuttle reactions, the Li_2_O manifests a notable extension of the first charge plateau through mechanisms. Qiao et al. coated the surface of NCM811 particles with Li_2_O through ball milling to obtain Li_2_O@NCM811 cathode material (**Figure** **3**a).^[^ ^47^ ^]^ The Li_2_O@NCM811 composite was synthesized by ball‐milling NCM811 with 20 wt% Li_2_O powder in an argon atmosphere, forming a uniform coating layer of 50 nm thickness as confirmed by TEM. To address the issue of O_2_ release, the decomposition mechanism of Li_2_O was further analyzed using in situ surface‐enhanced Raman spectroscopy. The peak corresponding to the elongation of O–O indicated the formation of $O_{2}^{-}$. Therefore, a fluorinated ether additive was incorporated into the electrolyte to prevent O_2_ formation. Due to its low reaction free energy, this additive preferentially undergoes a spontaneous nucleophilic reaction with $O_{2}^{-}$, which will inhibit further reactions that would lead to O_2_ release. Meanwhile, this nucleophilic reaction produces a protective film rich in LiF on the surface of the NCM811 cathode (Figure 3b), effectively enhancing the oxidation window voltage of the electrolyte. Consequently, an ether‐based electrolyte exhibiting high CE can be utilized for this system without concerns regarding cathode electrolyte interface side reaction.

Li_2_S demonstrates compatibility with various electrodes, positioning it as a potential cathode additive. Despite this advantage, several challenges persist in practical applications. For instance, the intermediate products resulting from the decomposition of Li_2_S are prone to react with commonly used carbonate electrolyte systems, and the low conductivity of Li_2_S suppresses the extraction of Li^+^.^[^ ^48^, ^49^, ^50^ ^]^ Sun et al. synthesized M/Li_2_S nanocomposites by mixing ultrafine Li_2_S powder with transition metal particles.^[^ ^51^ ^]^ Research has demonstrated that the delithiation mechanism of Li_2_S undergoes a conversion reaction involving the transition metals upon doping (Figure 3c). The compatibility between Li_2_S and carbonate electrolyte is facilitated by the stabilization of polysulfide intermediates through transition metals. The conversion reaction corresponds to the deintercalation and intercalation potential of lithium, which are below 3 and 2 V, fulfilling the requirement for lithium supplementation. Furthermore, within the composite material, dense metal particles enveloping the surface of Li_2_S effectively mitigate environmental side reactions. Liu et al. directly incorporated unpretreated Li_2_S microspheres into the LFP electrode material through ball milling.^[^ ^52^ ^]^ To mitigate the reaction between Li_2_S and the carbonate electrolyte, an ether‐based gel polymer electrolyte (GPE) with high ionic conductivity was employed to construct the battery system. The upper voltage limit of this ether‐based electrolyte reaches 4.6 V, effectively covering the operational voltage range for both LFP and Li_2_S.^[^ ^53^ ^]^ In the ether‐based electrolyte environment, the lithium polysulfide (LiPS) released from the decomposition of Li_2_S facilitates the formation of a robust inorganic SEI, which is rich in fluorine and contains substantial amounts of low valent sulfur at the anode interface (Figure 3d). This SEI plays a crucial role in inhibiting SEI side reactions and dendrite growth. The incorporation of 11.5 wt% Li_2_S into the LFP cathode resulted in a lithium supply of 160 ${\text{mAh g}}^{-1}$ (Figure 3e).

Li_3_N possesses an impressive theoretical specific capacity of up to 2308.5 ${\text{mAh g}}^{-1}$, indicating its potential as a lithium supplementation additive for cathodes. Nevertheless, several issues remain to be addressed in practical applications, including the excessively low decomposition potential and relatively poor stability in environments containing water.^[^ ^54^ ^]^ Park et al. directly mixed thoroughly ground Li_3_N powder with LiCoO_2_, the cathode material, to investigate the effect of Li_3_N as a cathode additive.^[^ ^55^ ^]^ Long cycle test results demonstrated that the electrode material with a small amount of added Li_3_N exhibited significantly enhanced cycle life. However, since the actual voltage platform for decomposing Li_3_N is ≈0.9 V, a portion of lithium was not released during initial charging cycles. Sun et al. investigated the role of crystalline Li_2_O passivation layers in enhancing the environmental stability of Li_3_N.^[^ ^56^ ^]^ By grinding and annealing Li_3_N in a glove box containing trace amounts of O_2_, a thin and dense passivation layer was formed on the particle surface, thereby achieving stability under ambient conditions. The electrochemical tests indicated that due to the presence of the passivation layer, the actual decomposition potential of Li_3_N increased from ≈0.9 to 4.3 V. Park et al. investigated the incorporation of Li_3_N into NCM to develop cathode materials for lithium free all‐solid‐state batteries.^[^ ^57^ ^]^ The findings revealed that the capacity retention rate of the pristine NCM electrode was merely 37.9% after 80 cycles, whereas the electrode material with an addition of 5% Li_3_N exhibited a significantly improved capacity retention rate of 75.8% after 200 cycles. This indicates a substantial lithium supplementation effect provided by Li_3_N.

Over the past few years, a wide variety of cathode additives have been developed to compensate for Li loss. However, their application in AFLMBs necessitates further investigation. For instance, Sun et al. addressed issues related to high decomposition potential and low ionic and electronic conductivity associated with LiF materials through transition metal doping methods.^[^ ^58^ ^]^ This approach successfully led to the development of a LiF/Co composite material with a high lithium supplementation capacity, which was effectively applied to cathode lithium supplementation in LIBs. Nevertheless, current applications of LiF in AFLMBs predominantly focus on modifying SEI.^[^ ^59^ ^]^ Besides, early cathode lithium supplementation additives such as Li_6_CoO_4_ and Li_5_FeO_4_ have not been extensively studied within the context of AFLMBs due to their limited lithium capacity and energy density reduction caused by decomposition residues.^[^ ^32^, ^60^, ^61^, ^62^ ^]^

### 2.3 Electrolyte Engineering for Lithium Replenishment and Reactivation

Electrolyte engineering primarily encompasses electrolyte modification and the use of electrolyte additives. Electrolyte modification regulates SEI formation and lithium‐ion mobility by altering the physicochemical properties of the electrolyte. This indirectly influences the CE of the battery system but generally cannot directly achieve lithium replenishment. In contrast, electrolyte additives influence the battery system by dissolving various substances into the electrolyte. By incorporating redox shuttles or lithium‐containing solvents, these additives can compensate for irreversible capacity loss.

Zhang et al. achieved the recovery of dead lithium generated during cycling by introducing 2,2,6,6‐tetramethylpiperidine‐1‐oxyl (TEMPO), a redox shuttle additive, into the electrolyte.^[^ ^63^ ^]^ The incorporated TEMPO reactivates dead Li into active lithium ions through reactions (3) and (4), enabling their reintegration into the electrode system. However, this approach is only effective for electrically isolated dead lithium and shows limited efficacy against irreversible capacity loss caused by SEI side reactions. Additionally, the inclusion of TEMPO introduces extra energy consumption during battery charging processes.

(3) $$\text{TEMPO}-e^{-}\to {\text{TEMPO}}^{+}$$

(4) $${\text{TEMPO}}^{+}+\text{Li}\to {\text{Li}}^{+}+\text{TEMPO}$$

**Figure 4:**
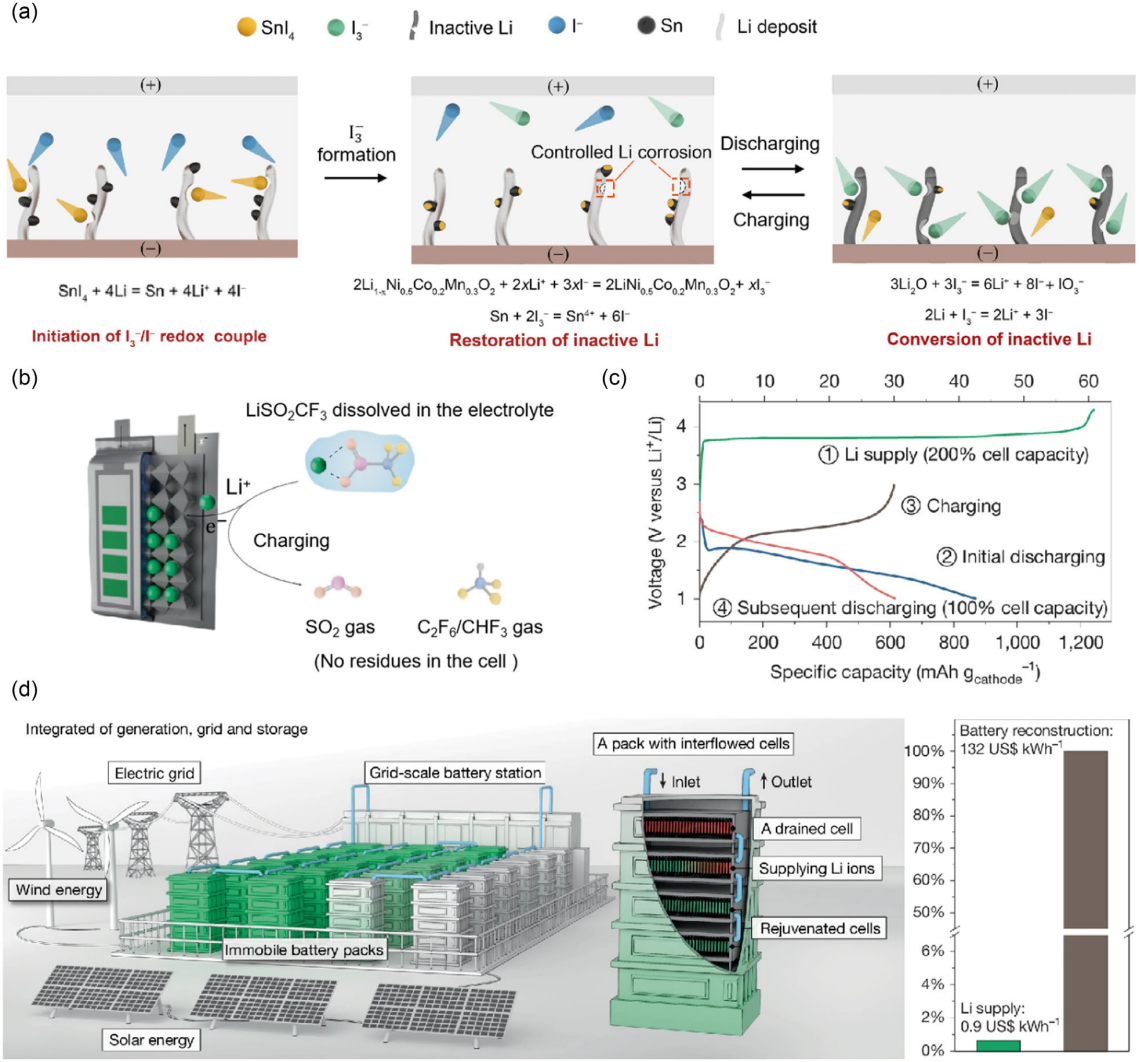
a) Schematic illustration of the principle of SnI_4_ electrolyte additive. Reproduced with permission.^[^ ^65^ ^]^ Copyright 2021, Wiley‐VCH. b) Schematic illustration of the lithium supplementation mechanism of LiSO_2_CF_3_ electrolyte additive. c) Voltage profile of a 388 ${\text{Wh kg}}^{\text{−1}}$ anode‐free pouch cell that incorporated an organic sulfurized polyacrylonitrile cathode with the LiSO_2_CF_3_ serving as the lithium supply. d) Envisioned grid energy storage based on external Li supply technique. Reproduced with permission.^[^ ^68^ ^]^ Copyright 2025, Springer Nature.

Jin et al. have restored the dead lithium and degraded SEI formed during the cycling process by introducing $I_{3}^{-}$ into the electrolyte, which partially alleviate irreversible capacity loss.^[^ ^64^ ^]^ The iodine‐containing electrolyte will facilitate the Li_2_O in the degraded SEI and dead lithium that had lost electrical contact back into active ${\text{Li}}^{+}$through reactions (5), (6), and (7). During charging, the $I^{-}$ produced from these reactions were subsequently re‐oxidized to $I_{3}^{-}$ at the cathode, thereby continuing this cycle (**Figure** **4**a). Although $I_{3}^{-}{\text{/I}}^{-}$ require migration through diffusion in the electrolyte, which reduces dead lithium recovery efficiency, they exhibit superior capability compared to TEMPO by additionally reclaiming Li_2_O from the SEI layer.

(5) $$3{\text{Li}}_{2}O+3I^{-}\to 6{\text{Li}}^{+}+{\text{IO}}_{3}^{-}+8I^{-}$$

(6) $$6\text{Li}+{\text{IO}}_{3}^{-}\to I^{-}+3{\text{Li}}_{2}O$$

(7) $$2\text{Li}+I_{3}^{-}\to 2{\text{Li}}^{+}+3I^{-}$$

While the $I_{3}^{-}$ converting the dead lithium, it also inevitably corrodes the lithium deposition layer on the anode. For this reason, Jin et al. used ${\text{Sn}}^{\text{4+}}$as the carrier of $I^{-}$, which finally achieved the protection of the deposited lithium layer through the sacrificial regulation of ${\text{Sn}}^{\text{4+}}$.^[^ ^65^ ^]^ On this basis, Wen et al. improved the lithium recovery efficiency of the redox couple by incorporating a high‐concentration LiNO_3_ additive.^[^ ^66^ ^]^ Further, Dong et al. modified this principle by utilizing a carbon paper (CP) current collector that is adsorbed with SrI_2_ for AFLMBs.^[^ ^67^ ^]^ The $I^{-}$ released into the electrolyte continuously restore dead lithium during the cycling process in accordance with the aforementioned principle. Meanwhile, ${\text{Sr}}^{\text{2+}}$ contributes to the formation of SEI in the form of SrF_2_, which mitigates SEI side reactions. Ultimately, the NCM523|CP@SrI_2_ AFLMB achieved a specific capacity of 129.1 ${\text{mAh g}}^{-1}$ over 200 cycles. However, restoring dead lithium does not directly introduce additional lithium into the electrolyte but rather enhances cycle life by saving existing lithium. Chen et al. employed machine learning techniques to identify the organic lithium salt LiSO_2_CF_3_ as an electrolyte lithium supplement, which offers comprehensive advantages across five key aspects: electrochemical activity, byproducts, specific capacity, solubility, and decomposition potential.^[^ ^68^ ^]^ During the initial charging cycle, the ${\text{SO}}_{2}{\text{CF}}_{3}^{-}$ undergo oxidation at the anode, resulting in the generation of SO_2_ and C_2_F_6_ gases that are subsequently released into the atmosphere. Meanwhile, ${\text{Li}}^{+}$ remains within the electrolyte as active lithium (Figure 4b). SO_2_ and C_2_F_6_ evolution peaks were detected by differential electrochemical mass spectrometry, confirming the decomposition of LiSO_2_CF_3_. Utilizing this material, AFLMBs were constructed with Cr_8_O_21_ and sulfurized polyacrylonitrile serving as cathode materials, respectively. High energy densities of 1192 and 388 ${\text{Wh kg}}^{-1}$ were achieved for these configurations. Additionally, the cycle life of the battery utilizing sulfurized polyacrylonitrile reached 440 cycles (Figure 4c). Besides, they also explored the application of this electrolyte additive in the field of battery recycling. The battery recycling and regeneration cost based on the electrolyte additive was greatly reduced (Figure 4d), indicating that this technology has broad application prospects in multiple fields.

The three lithium supplementation methods mentioned above demonstrate varying practical performances across different battery environments. **Table** **1** offers a detailed summary and comparison of their full cell cycling performances as documented in the literature.

**Table 1:** Comparison of the cycling performance of full cell with various lithium supply.

Cathode materiala)	Electrolyte	Current collector	Areal capacity [mAh cm^−2^]	Voltage window [V]	Rate [C]	Cycle	Capacity retention [%]	Energy density [Wh kg^−1^]	CE [%]	Ref.
Li_2_NMO	7 M LiFSI in FEC	Cu	3.2	3.3–4.8	0.1/0.2	50	88	367	95	[40]
Li_1+x_NCM811	6 M LiFSI in DME	Cu	6.4	2.2–4.3	0.1/0.2	100	84	447	99.83	[43]
Li_2_O@NCM811	1 M LiTFSI and 1.5 M LiFSI in G_3_ with 10 vol% of HFE	Cu	4.9	2.8–4.4	0.5	300	80	320	99.79	[47]
Li_2_S@LFP	GPE with 1.0 M LiTFSI in DOL/DME	Cu	3.7	2.5–3.8	0.2	400	80	300	99.6	[52]
Li_3_N@NCM	LPSC	In	1.2	1.8–3.7	0.2	200	75.8	–	–	[57]
NCM523	2 M LiFSI in DME with 2 [wt%] LiNO3 + 0.1 [wt%] TEMPO	Cu	1.9	3.0–4.3	1	75	71.8	–	99.5	[63]
LFP	LiPF_6_ in EC‐DMC with 5 [vol%] FEC, 5 [vol%] G_4n_ and 0.01[mM] I_2_ into LP30	Cu	1.4	2.5–3.8	0.1/0.5	20	80	–	–	[66]
NCM523	1 M LiPF_6_ in FEC/DEC (1:1 [vol%])	CP@SrI_2_	3.9	2.7–4.3	0.5	200	82	–	–	[67]
NCM523	1 M LiPF_6_ in FEC/DEC (1:1 [vol%]	Cu@SrI_2_	3.5	2.7–4.3	0.5	20	80	–	–	[67]
LFP	1 M LiPF_6_ in FEC/DEC (1:1 [vol%])	CP@SrI_2_	3.2	2.5–4.2	0.5	100	98	–	–	[67]
Organic sulfurized polyacrylonitrile	1 M LiTFSI in DOL/DME with 4 [%] LiNO_3_	CNT	8.7	1.0–2.6	0.2/0.5	440	80	388	–	[68]
LRM	1M LiPF_6_ in EC/DMC/TTE (1:1:1 [vol%])	Cu@PDS‐Li	0.8	2.0–4.8	1	100	57	–	95	[82]
LFP	1 M LiDFOB + 0.2 M LiBF_4_ in FEC/DEC (1:2 [vol%]), LRS separator	Cu	1.5	2.5–3.9	0.3	50	88.9	–	–	[83]
NCM811	1.0 M LiPF_6_ in EC/DEC/DMC (1:1:1 [vol%]), Li_2_S@C|PE separator	Cu@Ag	2.0	3.0–4.3	0.5	100	88	450	99.8	[84]

a) Abbreviations: Li_2_NMO: Li_2_Ni_0.5_Mn_1.5_O_4_; NMC532: LiNi_0.5_Mn_0.3_Co_0.2_O_2_; NCM811: LiNi_0.8_Mn_0.1_Co_0.1_O_2_; LFP: LiFePO_4_; FEC: fluoroethylene carbonate; DME: 1,2‐dimethoxyethane; LPSC: Li_6_PS_5_Cl; LiTFSI: lithium bis((trifluoromethyl)sulfonyl)azanide; LiFSI: lithium bis(fluorosulfonyl)imide; G_3_: triethylene glycol dimethyl ether; HFE: fluorinated ether additive 1,1,2,2‐tetrafluoroethyl 2,2,3,3‐tetrafluoropropyl ether; GPE; DOL: 1,3‐dioxolane; LiPF_6_: lithium hexafluorophosphate; DEC: diethyl carbonate; EC: ethylene carbonate; DMC: dimethyl carbonate; G_4n_: Li compatible tetraglyme with n M LiNO_3_; LP30: 1 M LiPF_6_ in EC and DMC (1:1, wt.); TEMPO: 2,2,6,6‐tetramethylpiperidine‐1‐oxyl; TTE: 1,1,2,2‐tetrafluoroethyl‐2,2,3,3‐tetrafluoropropyl ether; LRS: lithium replenishment separator; LRM: Li‐rich manganese‐based cathode of Li_1.2_Mn_0.54_Ni_0.13_Co_0.13_O_2_; PE: polyethylene; LiDFOB: lithium difluoro(oxalato)borate ; LiBF_4_: lithium tetrafluoroborate.

## 3 Challenges and Opportunities

**Figure 5:**
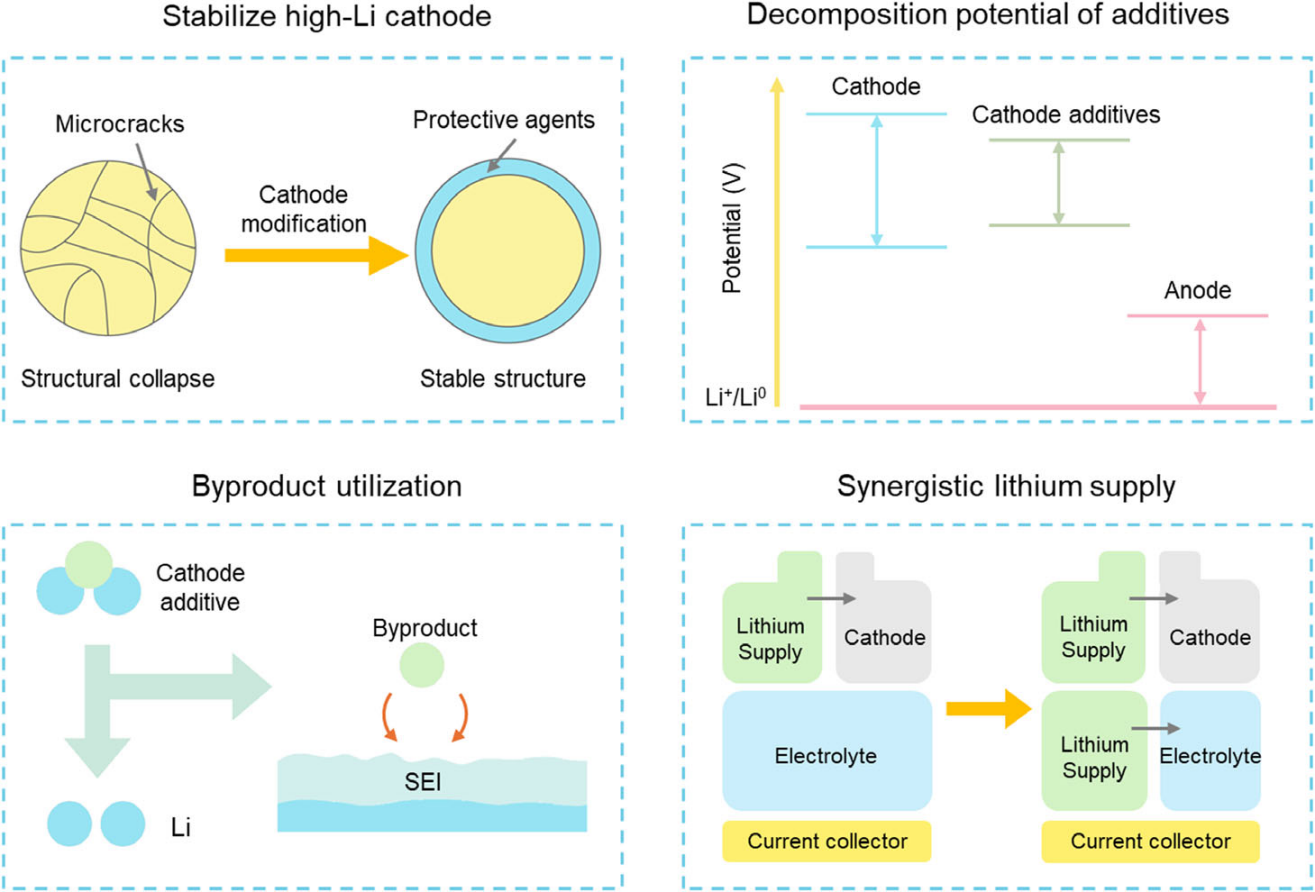
Schematic illustration of the challenges and opportunities associated with lithium supplementation. Stabilize high‐Li cathode by protective agents. Convert byproducts into beneficial components. Decomposition potential requirement of additives. Synergistic lithium supply of cathode and electrolyte.

While significant progress has been made in the development of lithium supplementation and restoration strategies for AFLMBs, several key challenges remain. These include issues related to material stability, electrochemical compatibility, and limited lithium compensation efficiency. At the same time, emerging approaches have provided new possibilities for enhancing battery performance. Therefore, balanced assessment of both challenges and opportunities is essential for guiding future research and practical implementation (**Figure** **5**).

Overlithiation is fundamentally based on existing cathode materials for lithium supplementation. However, not all cathode materials can achieve overlithiation. They must exhibit minimal volume change in the overlithiated state and should not induce irreversible structural damage, thereby ensuring prolonged cycle life following delithiation. Currently, LFP and NCM are the predominant commercial cathode materials used in lithium batteries. Among these, the layered‐structured ternary NCM cathode has developed specific overlithiation strategies, whereas olivine‐structured LFP has not yet seen an established overlithiation strategy. This lack is largely attributed to the inherently limited capacity of LFP, which constrains its application potential. For future research, though studies have been conducted on the overlithiation of NCM in existing literature, there remains a significant gap between current findings and the theoretical overlithiation limit (Li_2_NCM). This is primarily attributed to the excessive volume changes induced by lithium capacity exceeding 1.4, which leads to the pulverization of the cathode. To mitigate this issue, a protective shell layer based on Li_2_NCM can be developed to restrict excessive volume fluctuations through an external framework.

Cathode additives alleviate some of the inherent limitations of the cathode materials themselves. However, since these additives are integrated into the cathode structure, the compatibility issues with the original electrochemical reactions must be addressed. Currently, Li_2_O, Li_2_S, and Li_3_N represent several promising cathode additives that have demonstrated successful application cases. According to current research, the fundamental principles for designing cathode additives can be summarized as follows: 1) The lithium storage capacity of the additive should exceed that of the original cathode material, thereby providing additional lithium without increasing mass or volume. 2) The lithium supplementation material must release enough Li^+^ within the standard cut‐off voltage range. 3) It is essential that the lithium supplementation material does not undergo ${\text{Li}}^{+}$ intercalation during subsequent discharge cycles. Specifically, the onset voltage for the lithium intercalation reaction should remain below 2.5 V. 4) The chosen lithium supplementation material should exhibit minimal adverse reactions with the battery system to ensure normal operational functionality. This implies that it possesses a relatively high open‐circuit voltage. 5) Efforts should be made to minimize residual substances to enhance overall energy density.

Based on these principles, materials with relatively high lithium content have been extensively developed. New cathode additive materials are unlikely to surpass Li_2_O, Li_2_S, and Li_3_N in terms of lithium storage capacity. Consequently, the significance of further developing novel materials appears limited. However, there remains considerable potential for enhancing the research on existing cathode additive materials. The intermediate products resulting from the decomposition of cathode additives can be effectively utilized and transformed into beneficial components within the battery system. For instance, $O_{2}^{-}$ generated from the decomposition of Li_2_O facilitates the formation of a LiF‐SEI. Thus, investigating the decomposition mechanisms of cathode additives and converting their byproducts into substances advantageous to battery performance represents a promising avenue for future research. Many cathode additives currently employed in LIBs have yet to be explored in AFLMBs. The structural similarities between the cathodes of these two types of batteries support this potential application. However, numerous differences still necessitate continued investigation.

Electrolyte additives take both the restoration of dead lithium and the direct lithium supply into account. For those electrolyte additives that focus on restoring dead lithium, methods for rejuvenating byproducts other than Li_2_O within the SEI still necessitate further research. For instance, Xia et al. utilized the asymmetric lithium salt, lithium 1,1,1‐trifluoro‐N‐[2‐[2‐(2‐methoxyethoxy)ethoxy)]ethyl] methanesulfonamide (LiFEA) to dissolve organic components like ROCO_2_Li from the SEI into the electrolyte, promoting the formation of a dense and stable SEI.^[^ ^69^ ^]^ However, the presence of ROCO_2_Li in the electrolyte adversely affects lithium‐ion mobility. Exploring methods to revert such organic lithium species back into active lithium would not only further compensate for irreversible capacity loss but also enhance lithium‐ion mobility.

As for the lithium supplementation additives, while machine learning has been employed to identify theoretically optimal additives, there remains significant potential for research into the application of nonlithium cathode materials in AFLMBs. When the electrolyte assumes primary responsibility for supplying lithium, a broader range of nonlithium cathode materials can be utilized to enhance cycle life. In future developments, it is imperative to consider battery capacity with respect to both electrolyte and electrode components. By fostering synergy between electrolyte and electrode, optimization of capacity and cycle life can be effectively achieved.

In addition to lithium supplementation techniques, methods such as anode current collector modification and electrolyte engineering are also widely employed to enhance the cycling performance of AFLMBs. Current collector engineering is systematically categorized into interfacial engineering and bulk structural reconstruction. Interfacial engineering primarily enhances lithiophilicity through the strategic implementation of lithium‐alloying interphases and artificially constructed SEI layers.^[^ ^70^, ^71^, ^72^, ^73^, ^74^ ^]^ Bulk structural reconstruction leverages the intrinsic physicochemical properties of collector materials to facilitate homogeneous lithium deposition within 3D conductive scaffolds.^[^ ^75^, ^76^ ^]^ Concurrently, electrolyte design focuses on the optimization of solvation structures and fundamental physicochemical properties to achieve synergistic improvements in energy density and interfacial stability.^[^ ^77^, ^78^, ^79^, ^80^, ^81^ ^]^ **Table** **2** presents the performance of recently reported AFLMBs with modified current collector or electrolyte. Recently, multifunctional modification strategies have gained significant attention. Peng et al. constructed an artificial SEI composed of polydiallyl lithium disulfide (PDS‐Li) via in situ electrochemical synthesis. This approach delivers a dynamic lithium compensating capability while simultaneously improving interfacial compatibility.^[^ ^82^ ^]^ Similarly, lithium reservoir functionalized separators have been developed.^[^ ^83^, ^84^ ^]^ By combining multiple approaches that concurrently address lithium supplementation, current collector, and electrolyte performance, further improvements in the performance of AFLMBs are anticipated.

**Table 2:** Comparison of the cycling performance of recently reported AFLMBs.

Cathode materiala)	Electrolyte	Current collector	Areal capacity [mAh cm^−2^]	Voltage window [V]	Rate [C]	Cycle	Capacity retention [%]	Energy density [Wh kg^−1^]	CE [%]	Ref.
LFP	1 M LiTFSI in DOL/DME (4:1 [vol%]) with 3 [wt%] LiNO_3_	BSP‐Cu	2.1	2.0–3.8	0.3/0.5	200	70	–	98.5	[71]
NCM811	1 M LiBF_4_ + 1 M LiDFOB in tFEP/FEC	Cu	4.64	3.0–4.6	0.1/0.5	100	80	442.5	–	[72]
LFP	1 M LiTFSI in DOL/DME, (4:1 [vol%]) with 3 [wt%] LiNO_3_	DNA‐Cu	3.3	2.0–3.8	0.3/0.5	400	46.4	512	99.1	[73]
NCM811	1 M LiPF_6_ in EC/DEC with 10 [%] FEC + 1 [%] VC	d‐CP	4.2	2.7–4.3	0.5	50	90	349	–	[75]
NCM811	1 M LiPF_6_ in EC/DMC (1:1 [vol%])	BTO‐Cu	1.0	3.0–4.3	0.3	70	–	–	99.37	[76]
NCM523	1M LiPF_6_ in EC/DEC (1:1 [vol%]) with 5 [wt%] Sn(Oct)_2_	Cu	3.3	3.0–4.3	0.2/0.5	50	68	–	99.1	[77]
NCM333	LLZTO solid‐state electrolyte	TiN NTs	3.2	2.8–4.3	0.3	600	78.3	–	99.8	[78]
NCM333	1M LiPF_6_ in EC/DEC (1:1 [vol%]) with 2 [wt%] KPF_6_ + 2 [vol%] TMSP	Cu	1.69	2.5–4.3	0.1	20	48	–	95.21	[79]
NCM333	1M LiPF_6_ in FEC/TTE (3:7 [vol%])	Cu	1.65	2.5–4.5	0.25	65	50	–	98.67	[80]
NCM523	1M LiDFOB + 0.05M LiPF_6_ in FEC/TTE/DEC (2:2:1 [vol%])	Cu	2.03	2.5–4.3	0.1	35	45.4	–	98.07	[81]

a) Abbreviations: NMC532: LiNi_0.5_Mn_0.3_Co_0.2_O_2_; NCM811: LiNi_0.8_Mn_0.1_Co_0.1_O_2_; LFP: LiFePO_4_; NMC333: LiNi_0.33_Mn_0.33_Co_0.33_O_2_; BTO: BaTiO_3_; d‐CP: defective CP; BSP: supramolecular polymer; DOL: 1,3‐dioxolane; DME: 1,2‐dimethoxyethane; Sn(Oct)_2_: stannous octoate; tFEP: ethyl 3,3,3‐trifluoropropanoate; LiTFSI: lithium bis((trifluoromethyl)sulfonyl)azanide; LiPF_6_: lithium hexafluorophosphate; KPF_6_: potassium hexafluorophosphate; DEC: diethyl carbonate; EC: ethylene carbonate; FEC: fluoroethylene carbonate; DMC: dimethyl carbonate; VC: vinylene carbonate; TEMPO: 2,2,6,6‐tetramethylpiperidine‐1‐oxyl; TTE: 1,1,2,2‐tetrafluoroethyl‐2,2,3,3‐tetrafluoropropyl ether; PE: polyethylene; LiDFOB: lithium difluoro(oxalato)borate; LiBF_4_: lithium tetrafluoroborate; TMSP: trimethylsilyl phosphate; LLZTO: Li_7_La_3_Zr_1.5_Ta_0.5_O_12_.

The practical implementation of lithium supplementation strategies in the fabrication of AFLMBs faces significant challenges in cost and scalable manufacturing for industrial deployment. The lithium supplementation process necessitates additional materials and processing steps, increasing production costs. Furthermore, scaling up production introduces complications such as impurity removal and processing homogeneity issues, which collectively compromise the effectiveness of lithium compensation.^[^ ^85^ ^]^ Consequently, despite promising laboratory‐scale results, the industrial viability of these strategies requires rigorous evaluation through manufacturing practices and further improvements.

## 4 Conclusion

This perspective provides a concise summary of recent advancements in lithium supplementation technologies for AFLMBs, aiming to elucidate various design principles and offer insights into future development prospects. Lithium supplementation technologies have emerged as a pivotal method for mitigating the irreversible capacity loss of AFLMBs. Currently, the predominant methods developed for lithium supplementation encompass overlithiation, cathode additives, and electrolyte additives. The representative material research of each classification is summarized in this perspective. As these technologies continue to evolve, a notable trend has emerged that decouples the lithium supplementation process from the constraints imposed by cathode materials. Significant progress toward this trend has been facilitated by advancements in electrolyte additives. Synergistic lithium supplementation that integrates both electrolytes and electrodes represents a balanced approach to enhancing energy density and cycling performance, thus indicating a promising direction for further advancement.

## Conflict of Interest

The authors declare no conflict of interest.
